# Mapping Sensory Spots for Moderate Temperatures on the Back of Hand

**DOI:** 10.3390/s17122802

**Published:** 2017-12-04

**Authors:** Fan Yang, Guixu Chen, Sikai Zhou, Danhong Han, Jingjing Xu, Shengyong Xu

**Affiliations:** Key Laboratory for the Physics and Chemistry of Nanodevices, and Department of Electronics, Peking University, Beijing 100871, China; fyang1992@pku.edu.cn (F.Y.); chenguixu@pku.edu.cn (G.C.); zhousikai@pku.edu.cn (S.Z.); danhonghan@pku.edu.cn (D.H.); xujj@pku.edu.cn (J.X.)

**Keywords:** thermosensation, sensory spots, distribution, electronic skin

## Abstract

Thermosensation with thermoreceptors plays an important role in maintaining body temperature at an optimal state and avoiding potential damage caused by harmful hot or cold environmental temperatures. In this work, the locations of sensory spots for sensing moderate temperatures of 40–50 °C on the back of the hands of young Chinese people were mapped in a blind-test manner with a thermal probe of 1.0 mm spatial resolution. The number of sensory spots increased along with the testing temperature; however, the surface density of sensory spots was remarkably lower than those reported previously. The locations of the spots were irregularly distributed and subject-dependent. Even for the same subject, the number and location of sensory spots were unbalanced and asymmetric between the left and right hands. The results may offer valuable information for designing artificial electronic skin and wearable devices, as well as for clinical applications.

## 1. Introduction

Recently, electronic skin (e-skin) integrated with a variety of sensors has been developed for wearable devices [1] and health monitoring [2,3]. Among these electronic components in e-skin, temperature sensors are an important element, since temperature is a critical parameter of living organisms. In nature, a biosystem senses internal and/or external temperature (T) through thermoreceptors. Warm and cold receptors in humans, which are sensitive to hot and cold stimulus, respectively, can discriminate temperatures ranging from −10.0 °C to 60.0 °C [4]. The thermosensation of skin may provide regulated input signals for maintaining the body temperature at an optimal constant state, and it may also sense potentially damaging temperature stimulus, thus being helpful to protecting the structure and function of the human body [5]. Many related issues at the molecular level have been investigated [6,7,8]. It has generally been accepted that the transient receptor potential (TRP) channels, which exist in membranes of cells, nerve endings or intracellular organelles, serve as thermoreceptors in thermosensation [9]. Among them, TRPV1, TRPV2, TRPV3, and TRPV4 are recognized as the main TRPs for warm sensation [10,11], and TRPM8 and TRPAl are known to take charge of cold sensation [10,12]. Some thermoreceptors show more complicated functions. For example, TRPV1 can be activated not only by heat (T ≥ 43.0 °C) [13,14], but also by chemical stimulus, such as capsaicin [15,16] or change in pH [17,18]. TRPV2 is found activated at temperatures higher than 52.0 °C, therefore it plays an critical role in the defense system of the human body [19]. The activating thresholds at warm temperatures for TRPV3 and TRPV4 are in the ranges of 33.0–39.0 °C [20,21] and 27.0–34.0 °C [22,23], respectively. TRPM8 and TRPA1 are cold activated channels corresponding to the moderately cool temperatures of 23.0–28.0 °C [24] and harmful cold temperatures below 17.0 °C [25], respectively. These TRPs are nonselective cationic channels and widely expressed in *A_δ_*- and *C*- fibers [26,27]. Under different activating temperatures, they generate corresponding signals with the internal flow of calcium ions [13].

Although the exact working mechanism of TRPs is not clear, detailed information on the distribution of warm or cold sensory spots on skin is still valuable for designing artificial e-skin. The location of sensory spots is supposed to directly indicate the regions which are rich with various warm or cold receptors underneath the epidermis. In 1927, based on earlier studies, Dallenbach carefully measured the warm and cold spots on the human hand, forearm and upper arm at 42.0–44.0 °C, and reported that the average numbers of warm spots ranged from 7.5/cm^2^ to 22.5/cm^2^ [28]. In 1934, Heiser et al. found that increasing stimulus-pressure resulted in a larger number of thermal-sensations on the forehead [29]. Later in 1995, Tamura et al. reported a warm spot density that ranged from 1 to 6/cm^2^ at 40 ± 0.5 °C for 10 female students over 25 regions, including the forehead, cheek, hand, and leg [30]. The measured warm or cold sensory spots in these experiments might correspond to receptors that consist of several kinds of TRPs. Yet to date, the details of these receptors and the exact locations of sensory spots over human skin, remain unclear. The missing information may offer valuable clues for studies on the distribution of different TRPs and the design of e-skin. In this work, we mapped the locations of sensory spots for moderate temperatures of 40–50 °C on the backside of the hands for Chinese college students. A small number of sensory spots were found, which were located irregularly with a remarkable difference among individuals and which occurred asymmetrically between the two hands of the same subject.

## 2. Materials and Methods

We have fabricated a special thermal probe for this work. The probe structure and experimental setup are illustrated in Figure S1 (see Supplementary Material). The core of the thermal probe, a copper rod, was heated with a 30 cm long resistance wire (OCr25AL5, resistivity 20.3 Ω/m) and a DC power supply (Longwell DC PS-1503D). The probe tip had a diameter of 0.5 mm. A small type-K thermocouple (TC) was embedded in the probe core at 2.5 mm away from the tip to monitor the tip temperature. The exact tip temperature was calibrated with the embedded TC at varied heating powers (Figure S1b), resulting in an error of ±0.3 °C for any temperature set points in 40–50 °C. During each measurement, the subject sat still in a relaxed state with eyes blocked by a pair of sleeping masks. When the thermal probe was operated in active mode, the tip temperature was kept at a set point between 40.0–50.0 °C. While in the passive mode, the heating power was cut off and the tip temperature was the same as the room temperature, 27.0 ± 1.0 °C. As illustrated in Figure S1c, one tester slowly moved the probe tip approaching the skin surface of the subject, and recorded the voice feedback of the test subject. As pre-agreed, when feeling a distinct hot sensation which was remarkably different from the tactile sensation, the subject gave a positive feedback by saying “hot”, otherwise he/she kept silent. Another tester was in charge of controlling the power and setting the tip temperature. This blind-test routine could efficiently prevent psychological hints or other factors from disrupting the experimental results. The laboratory was kept at an air humidity of 40 ± 5%. Before each measurement, the subjects were required to stay in the laboratory for at least 30 min to adjust themselves to a relaxed state.

The aim of the research is to learn about the locations of sensors in human, which is related to the working mechanism of the brain. However, since the human palm is sensitive to tactile sense, it is difficult for people to distinguish thermal sense from tactile sense, especially at low testing temperatures. In addition, a long-time test sweeping on the palm with a metal probe can make subjects feel very uncomfortable. Hence, we chose the backside of hand as a typical area to perform the measurement. The backside surface of both hands of each subject were pre-marked with ink into square grids with size of 1.0 × 1.0 cm^2^, each contained 10 × 10 testing points, so that the special resolution was 1.0 mm. Each point was tested for two seconds, and the break time between measuring each point was also about two seconds. A two second testing time was long enough for the sensory spot to perceive the thermal signal. The probe was swept point-by-point by the tester over the whole testing area.

In the first round of experiments, four Chinese college students, referred to as ***α***, ***β***, ***γ***, and ***δ***, served as the testing subjects. They all lived in regular routines and were in good health. Female subjects ***α***, ***β*** and male subjects ***γ***, ***δ*** who were 24, 25, 20, and 22 years old; had heights of 160, 158, 172, and 183 cm; and weights of 44, 50, 65, and 94 kg, respectively, were tested individually. All of them were right-handed. Under the same conditions, each subject was tested for three times at every temperature point with a time interval of one day to one week for statistics analysis.

In the second round of experiments, to verify the obtained results and further check the difference of locations of sensory spots between left-handed and right-handed people, 10 more students in good health, aged 21–33, were tested over four days. The five female subjects were referred to as F1, F2, F3, F4, and F5, and the five male subjects were referred to as M1, M2, M3, M4, and M5. Subjects F4, F5, M3, M4, and M5 were left-handed. They were tested only for 40.0 °C, 45.0 °C, and 50.0 °C. Their physical characteristics were listed in Table S1 (see Supplementary Material).

## 3. Results

**Figure 1:**
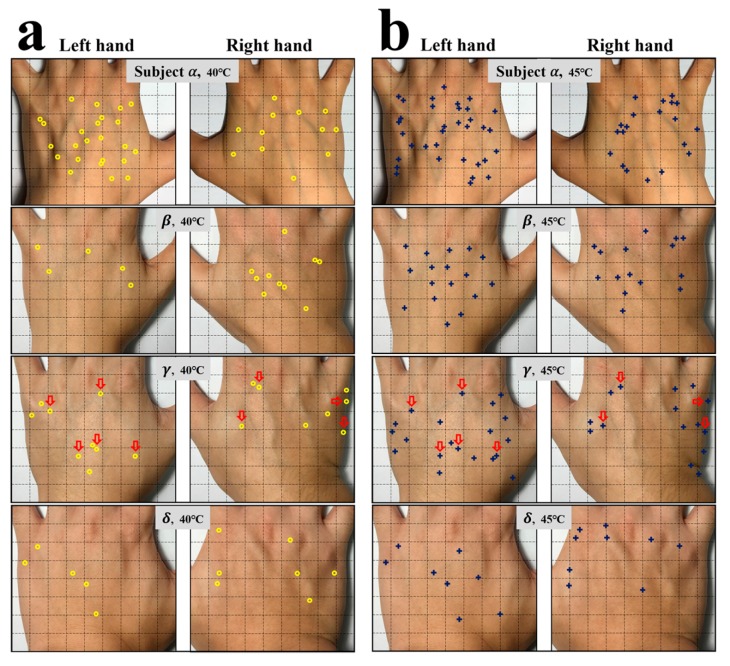
Photographs for the locations of sensory spots on the back of hands of subjects ***α****,* ***β****,* ***γ***, and ***δ***. (**a**) Yellow circles and (**b**) Blue crosses highlight the locations of sensory spots responded to at 40.0 and 45.0 °C, respectively. Red arrows in (**a**,**b**) are used to mark the locations of fixed sensory spots responded to at 40.0 and 45.0 °C.

Figure 1 presents photographs for the backsides of both left and right hands of the four subjects, ***α***, ***β***, ***γ***, and ***δ***. In the left panel (Figure 1a), the locations of sensory spots of one of the three same conditional measurements for the probe tip temperature of 40.0 °C are highlighted with yellow circles. In the right panel (Figure 1b), the locations of sensory spots of one of the three same conditional measurements for tip temperature of 45.0 °C are marked with blue crosses. The spatial resolution of sensory spots was about ±1 mm. During the measurement, we found that except most separated sensory spots, there existed several coupled sensory spots that were close to each other with a spacing of a few millimeters. In rare cases, in a small area of the skin, many points seem sensitive. This could be result from a cluster of sensory points. One sees a striking feature from these data, that the numbers of sensory spots on the back of the hands of these four subjects are very small. For the tip temperature of 40.0 °C, subject ***α*** only had 24 sensory spots on the backside of her left hand and 12 spots on the right. At the same temperature, these numbers for subject ***δ*** were even smaller, i.e., 5 and 7. For the tip temperature of 45.0 °C, the numbers of sensory spots on the left and right hands for subject ***α*** were 34 and 20, respectively, but those for subject ***δ*** were only 8 and 9, respectively. A second remarkable feature is that the sensory spots are located irregularly on the backside of each hand. For sensing 40.0 °C, for example, the sensory spots of subject ***α*** were scattered evenly on the whole of the left hand, while those for subject ***β*** occurred only on the upper part. More surprisingly, for the same subject the location and number of sensory spots on the left and right hands were not similar or balanced to each other. In the case of subject ***α***, for instance, the number of sensory spots for sensing 40.0 °C on her left hand was two times of that on her right hand, and their distribution patterns were quite different. A similar trend is observed in all of these four subjects.

**Figure 2:**
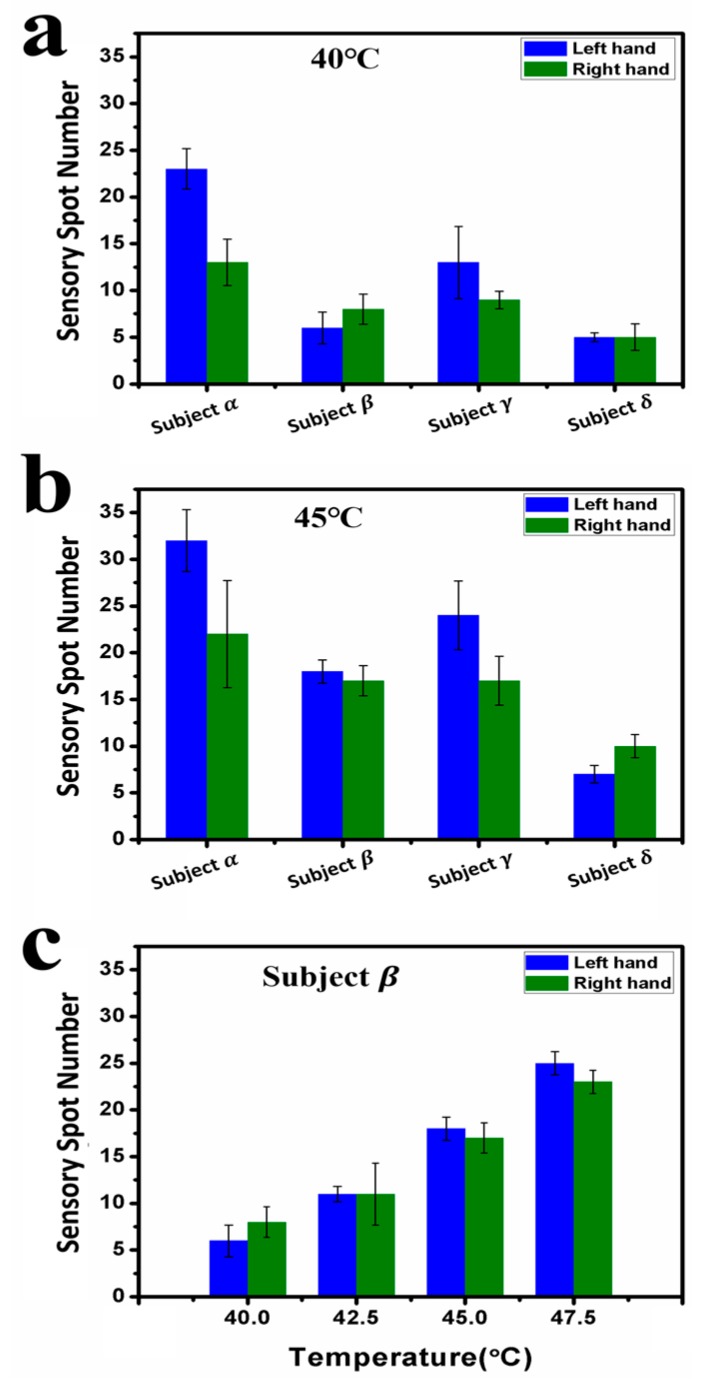
Statistical results of sensory spots on the back of the hands of subjects ***α****, **β**, **γ***, and ***δ***. Average numbers of sensory spots for four subjects responded to (**a**) at 40.0 °C and (**b**) at 45.0 °C, respectively. (**c**) Average numbers of sensory spots responded to at 40.0, 42.5, 45.0, and 47.5 °C for subject ***β***.

We have also found that the number of sensory spots increased with the testing temperature, as shown in Figure 2a,b, which plot the average testing results of the three same conditional measurements at 40.0 °C and 45.0 °C for all four subjects, respectively. The number of spots were not balanced between two hands. For 40.0 °C, subjects ***α*** and ***γ*** had more sensory spots on their left hand than the right, but subject ***β*** had more spots on her right hand than on the left, and subject ***δ*** had nearly equal number of spots on both hands. Figure 2c shows a typical result for subject ***β*** measured at 40.0, 42.5, 45.0, and 47.5 °C. The trend that the average numbers of sensory spots on both hands increase with the testing temperature can be clearly seen. Interestingly, only a small portion of the spots kept their locations when tested at varied temperatures. With increasing temperature, the newly measured sensory spots at higher temperatures often occurred at different locations. Comparing the distributions of sensory spots shown in Figure 1a,b, taking subject ***γ*** for an example, 5 of the 20 spots on his left hand and 4 of the 15 spots on his right hand at 45.0 °C (highlighted with red arrows) had the same locations with those measured at 40.0 °C.

**Figure 3:**
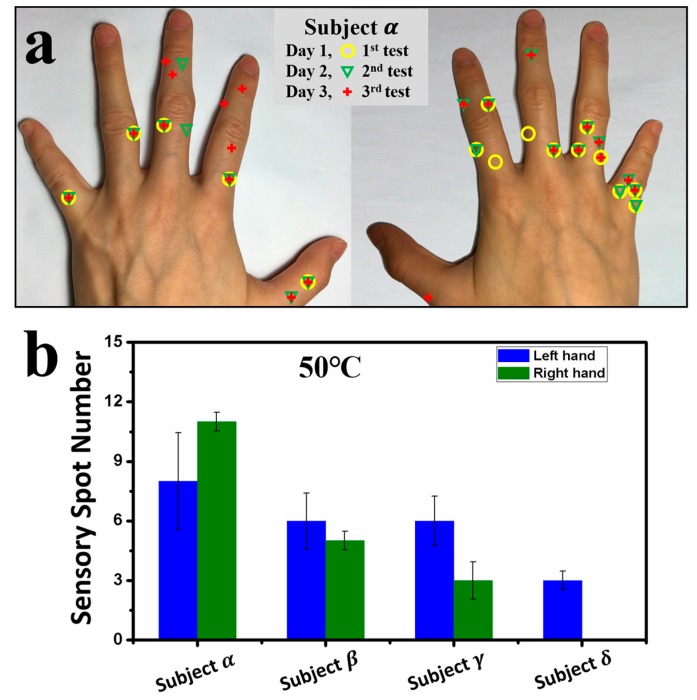
Results of locations and numbers of sensory spots on the backsides of the fingers of subjects ***α***, ***β***, ***γ***, and ***δ***. (**a**) Photographs for the locations of sensory spots responded to at 50.0 °C on the backsides of the fingers for subject ***α***. Yellow circles highlight the first test result, green triangles highlight the second test result, and red crosses highlight the third test result; (**b**) Average numbers of sensory spots responded to at 50.0 °C on the backsides of fingers for the four subjects.

When the probe tip temperature was increased to 50.0 °C, most areas on the back of the hand caused insufferable burning tingling sensation for all the subjects. However, for fingers, the 50.0 °C stimulating temperature was bearable. Figure 3a shows typical results for three runs of measurements taken over three days for the sensory spot distributions on the backside of the fingers for subject ***α***, where the locations of the three measurements are highlighted with different shapes and colors. Similarly, it shows a small number of spots and asymmetric distribution between the left and right hands. One sees that there are five fixed sensory spots for each hand, where the results of three runs overlap. In Figure 3b, the number of sensory spots on the fingers at 50.0 °C are summarized. For subjects ***β*** and ***γ***, they showed less sensory spots on the right fingers. For subject ***δ***, no sensory spot was recognized on his right fingers.

**Figure 4:**
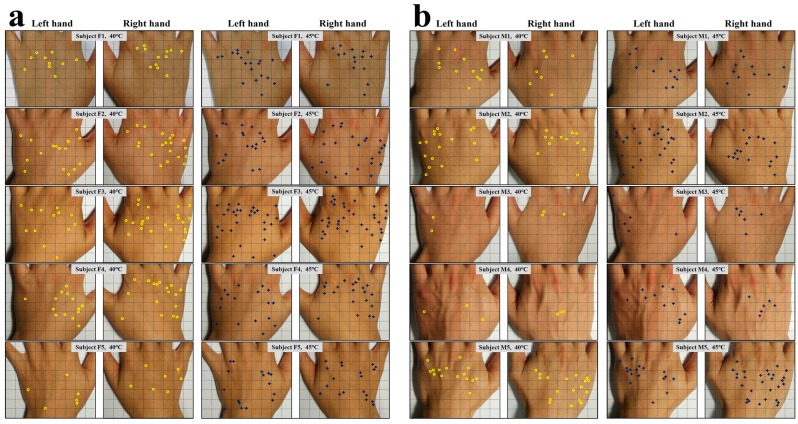
Photographs for the locations of sensory spots on the back of hands of 10 subjects. The locations of sensory spots of (**a**) the five female subjects and (**b**) the five male subjects responded to at 40.0 °C and 45.0 °C are highlighted by yellow circles and blue crosses, respectively.

Figure 4 shows the locations of sensory spots on the back of the hands of the 10 subjects tested in the second round of experiments. In the left panel (Figure 3a), the locations of sensory spots of five female subjects for the probe tip temperature of 40.0 °C and 45.0 °C are highlighted, and in the right panel (Figure 3b), the locations of sensory spots of five male subjects at corresponding testing temperatures are marked. From this figure, the small numbers of sensory spots and irregular locations and asymmetric distribution between the left and right hands of the same subject were repeatedly observed. The male subjects M3 and M4 even had only 2–3 sensory spots at 40.0 °C. Yet it is not clear what factors determine the number of sensory spots for each person.

**Figure 5:**
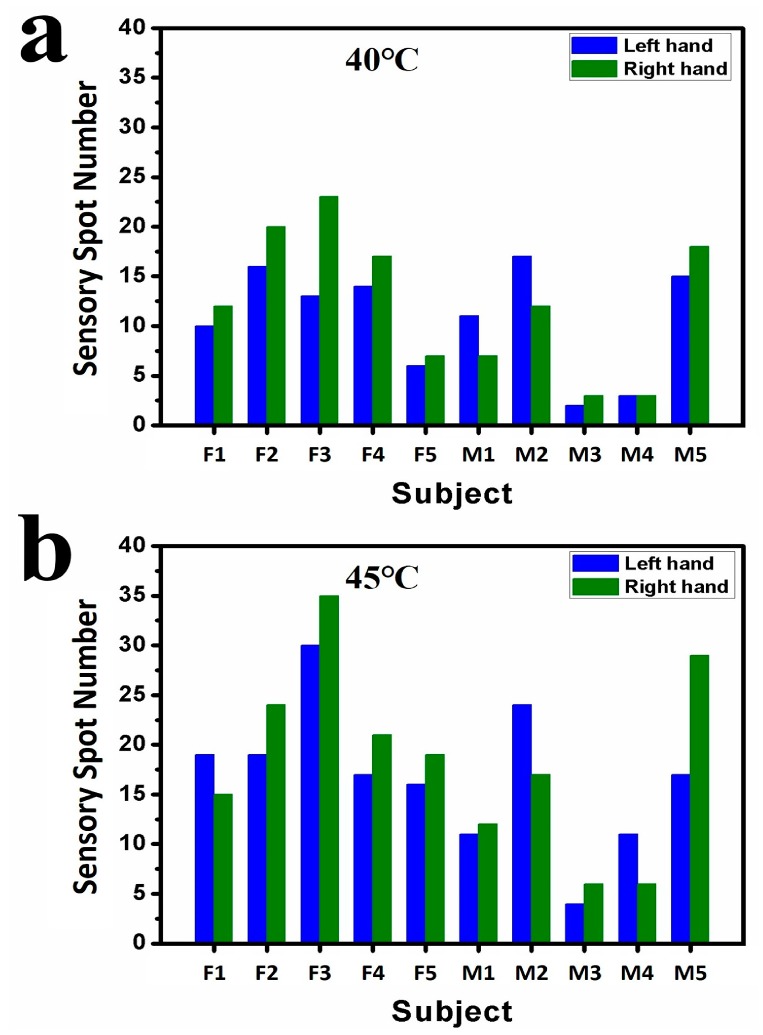

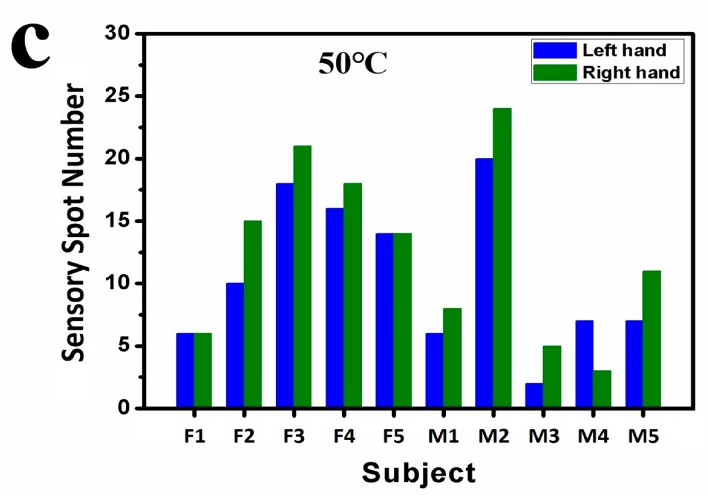
Numbers of sensory spots on the backsides of hands and fingers of 10 subjects in the second round of experiments. Numbers of sensory spots responded to (**a**) at 40.0 °C and (**b**) at 45.0 °C on the back of hands; (**c**) Numbers of sensory spots responded to at 50.0 °C on the backsides of fingers.

Figure 5 plots a statistics analysis of the testing results for the second round of experiments. All the five female subjects and left-handed male subjects M3 and M5 had more sensory spots on the right hands at 40.0 °C. Only right-handed male subjects M1 and M2 were found more sensory spots on their left hands. For 45.0 °C, subjects F2, F3, F4, F5, M1, M3, and M5 showed more sensory spots on their right hands, while subjects F1, M2, and M4 showed more sensory spots on their left hands. The trend that the number of sensory spots increases along with the testing temperature is obvious. For 50.0 °C, F2, F3, F4, M3, and M5 still had larger numbers of sensory spots on their right fingers and the largest number of sensory spots measured on the fingers in this round of experiments was 24, on the right fingers of subjects M2. From current data, no correlation was found between the locations of sensory spots and the habits of hand use.

## 4. Discussion

Compared with the data reported previously, the numbers and densities of sensory spot on the back of the hands of our subjects are remarkably smaller. For example, at 45.0 °C the highest sensory spot density obtained from subject ***α*** is about 8–15% of those reported previously [28,30]. This is not likely caused by measurement errors. One possible reason could be the selection of the testing area. Both Tamura and Dallenbach selected only an area of 2 × 2 cm^2^ for their measurements. We have measured the whole hand area for our subjects, e.g., 54 cm^2^ for ***α*** and 64 cm^2^ for ***δ***. As shown in Figure 1, high local density of sensory spot occurs at certain small areas. Another reason might lie in the subjects, who were of different races and health conditions.

The unbalanced number and asymmetric location of sensory spots between left and right hands for the same subject, a phenomenon repeatedly observed in all subjects, indicate interesting clues for the structure of the hand's nerve system. It has been reported that a variety of central nervous system (CNS) tissues and apparatus are the result of the neurogenic asymmetric division of neural epithelium (NE) cells [31]. In humans, this asymmetric phenomenon may occur during the embryonic development, yet it may be caused by other facts during the growth of these subjects. This needs further studies to make it clear.

The maps of sensory spots at different temperatures that we obtained should depict the distribution of different TRPs to a certain extent. Fixed locations of sensory spots at varied temperatures may indicate the coexistence of several types of TRPs, e.g., TRPV1 and TRPV3. The fact that the number of sensory spots increases with the testing temperature is understandable with the molecular mechanism for thermosensation [32]. The insufferable burning tingling sensation tested at 50.0 °C over the whole area of the back of the hands of the subjects might indicate a different mechanism. Probably at this and higher temperatures most TRPs—TRPV2 in particular—are simultaneously activated and therefore cause severe biochemical and bioelectrical reactions.

## 5. Conclusions

In summary, we have pinpointed the locations of sensory spot at temperatures of 40.0, 42.5, 45.0, 47.5, and 50.0 °C on the backsides of both left and right hands for young Chinese college students. The number of sensory spots, up to 35 for a single hand, is found to increase along with the testing temperature and varying remarkably among different subjects. The locations of the spots are irregular on each hand. Even for the same subject, the location and number of sensory spots are not similar or balanced between the left and right hands. Except for several fixed locations, the newly occurred sensory spots for higher temperatures appear at different locations. The results may offer clues for studies on the distribution of TRPs, the structure and function of nerve endings, and the embryonic development process.

To fully understand the underlying mechanism of the unique distribution of sensory spots on human skin, a large amount of measurement data from more subjects at different ages and different conditions of health are needed. Yet our preliminary results have revealed some important features of sensory spots, such as the very limited number and random distribution. They may offer a valuable clue for thermal therapies in clinic applications, as many reports have shown that heating thermally susceptible points can regulate the physiological state of the human body. These results may also offer reference for the design of novel e-skin and wearable devices.

## Supplementary Materials

The following are available online at www.mdpi.com/1424-8220/17/12/2802/s1, Figure S1: Illustration of measurement setup and principle for the locations of sensory spots. Table S1: Physical Characteristics of Subjects.
