# Supplementary material for: Mapping Sensory Spots for Moderate Temperatures on the Back of Hand
Source: Sensors (Basel). 2017 Dec 4;17(12):2802. doi: 10.3390/s17122802 (PMC5750673; doi:10.3390/s17122802)
Supplement: Supplementary file 1 [file sensors-17-02802-s001.pdf]

Supplementary Material

Mapping sensory spots for moderate temperatures on the back of hand

Fan Yang, Guixu Chen, Sikai Zhou, Danhong Han, Jingjing Xu, Shengyong Xu*

Key Laboratory for the Physics and Chemistry of Nanodevices, and Department of Electronics, Peking University, Beijing, 100871, P. R. China

* Correspondence: xusy@pku.edu.cn

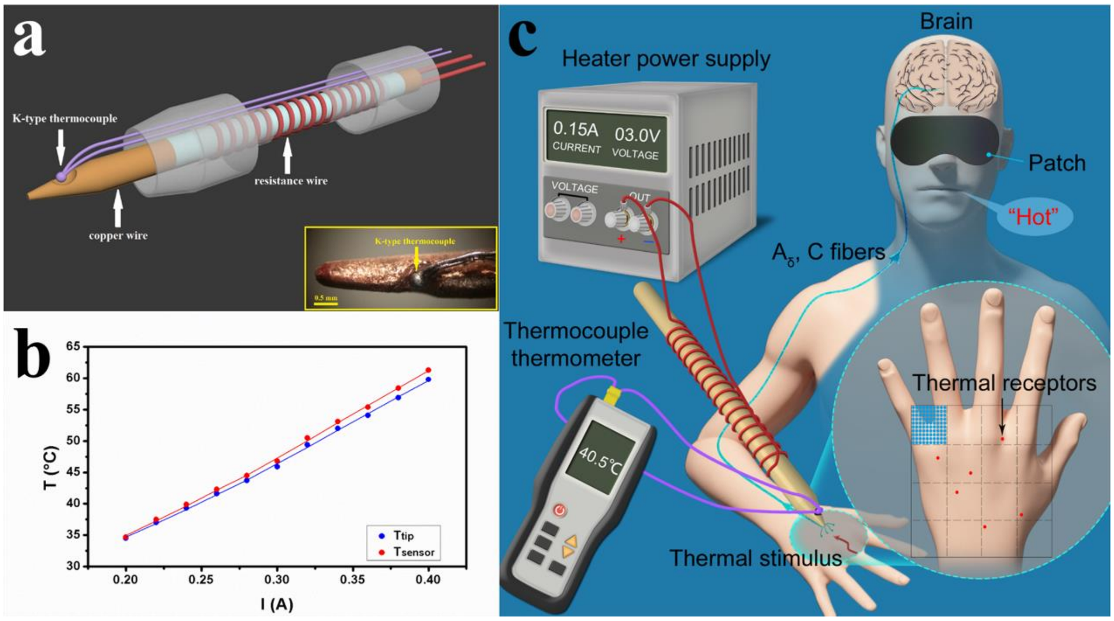

Supplementary Figure 1. Illustration of measurement setup and principle for the locations of sensory spots. **(a)** Illustration of the structure of thermal probe. It consists of a copper rod, a controllable resistance wire, a commercial K-type thermocouple. The inset microscope photograph shows the structure of probe tip in detail. **(b)** Temperature calibration curves between the probe tip site temperature of and sensor site temperature. Blue dotted line represents the relationship between the output current and the tip temperature. Red dotted line represents the relationship between the output current and the sensor site temperature. **(c)** Measurement principle for the locations of sensory spots on the back of hand with the blind-test. Thermal probe generates thermal stimulus, sensory spots sense thermal signals, then signals are transferred to the brain through A_δ- and C- fibers, subject says "hot" when she/he receives thermal signals.

Table S1 Physical Characteristics of Subjects

Subject	Age	Height (cm)	Weight (Kg)	Left-/Right-handedness	Temperature (℃)	
					Left Hand	Right Hand
**F1**	21	162	55	Right-handedness	33.9	33.6
**F2**	22	169	53	Right-handedness	34.8	35.2
**F3**	25	174	73	Right-handedness	34.8	34.7
**F4**	24	158	48	Left-handedness	34.4	34.3
**F5**	25	172	52	Left-handedness	34.2	33.7
**M1**	23	180	85	Right-handedness	34.8	34.9
**M2**	23	175	80	Right-handedness	34.7	34.5
**M3**	21	170	55.5	Right-handedness	33.0	33.2
**M4**	27	177	80	Left-handedness	34.4	34.6
**M5**	33	167	58	Left-handedness	33.7	33.6

Supplementary Table 1. The temperatures of subjects' hands were measured before tests. The temperature error was about ± 0.5 ℃.
